# Salivary Flow Rate in Patients with Sjögren’s Syndrome: Correlations with Salivary Gland Ultrasound Findings and Biomarkers of Disease Activity

**DOI:** 10.3390/ijms26010101

**Published:** 2024-12-26

**Authors:** Alexandru Caraba, Deiana Roman, Viorica Crișan, Stela Iurciuc, Mircea Iurciuc

**Affiliations:** 1 Second Department of Internal Medicine, “Victor Babes” University of Medicine and Pharmacy, 300041 Timisoara, Romania; caraba.alexandru@umft.ro; 2 Railway Clinical Hospital, 300041 Timisoara, Romania; iurciuc.stela@umft.ro; 3 Emergency Clinical Municipal Hospital, Rheumatology Department, 300041 Timisoara, Romania; viocrisan2002@yahoo.com; 4 Cardiology Department, “Victor Babes” University of Medicine and Pharmacy, 300041 Timisoara, Romania; iurciuc.mircea@umft.ro

**Keywords:** biomarkers, salivary flow rate, salivary gland ultrasound, Sjögren’s syndrome

## Abstract

Sjögren’s syndrome (SS) is a slowly progressive, chronic autoimmune inflammatory condition characterized by the affliction of the exocrine glands, with issues that derive from it markedly decreasing the quality of life of these patients. Salivary gland involvement can be identified through imaging methods. Among them, salivary gland ultrasonography (SGUS) is used as a diagnostic and prognostic tool in pSS. The aim of the present study was to assess the salivary flow rate and correlations between it and SGUS findings and markers of pSS activity. A total of 112 patients with pSS and 56 healthy subjects were included in this study. All patients underwent investigations including the measurement of serum autoantibodies, salivary flow rate determination, and ultrasonographic evaluation. SGUS modifications had a strong inverse correlation with salivary flow (r = −68.002, *p* < 0.0001) and a positive, strong correlation with IL-6 and Beta-2-microglobulin (r = −0.78 and r = −0.84, respectively, *p* < 0.001 in both cases). The SGUS findings were also strongly and positively correlated with the ESSDAI (r = −0.88, *p* < 0.0001) and Focus scores (r = −0.82, *p* < 0.0001). SGUS represents a non-invasive means of assessing the state of the salivary glands and, implicitly, the salivary flow of patients, offering valuable insights into disease progression and steps that can be taken in order to improve patients’ quality of life.

## 1. Introduction

Sjögren’s syndrome (SS) is a slowly progressive, chronic autoimmune inflammatory condition characterized by the affliction of the exocrine glands, while it is also associated with extra-glandular involvement. It is known as an autoimmune exocrinopathy [1]. The most common clinical manifestations are a consequence of the lacrimal gland involvement (keratoconjunctivitis sicca) and that of the salivary glands (xerostomia), which define the sicca complex [2]. SS is diagnosed mainly in the fifth and sixth decades of life, with the female sex being more frequently affected [3]. Depending on the association with other diseases or the lack thereof, SS is classified as primary or secondary Sjögren’s syndrome [4].

The epidemiology of primary Sjögren’s syndrome (pSS) varies throughout publications, with the overall consensus indicating an incidence of about 0.3–26.1/100,000 persons and a prevalence of about 13.1–32/100,000 persons. Both the incidence and prevalence show an increase with age [5].

One of the main issues found in patients with pSS is represented by xerostomia, which can prove to be increasingly problematic in more severe cases, leading to altered taste, difficulties in chewing, swallowing dry food, speaking for a prolonged period of time, and even the appearance of dental cavities and oral candidiasis [2]. All the pSS clinical manifestations are the consequence of the inflammatory process in both the exocrine glands and extra-glandular organs.

Histopathological studies of the salivary glands revealed that patients with pSS present high levels of inflammatory infiltrate that consisted of mainly CD4+ T cells, B cells (representing approximately 90% of the inflammatory cells), and in a smaller percentage, a combination of plasma cells, CD8+ T cells, FoxP3+ T regulatory cells, CD56+ natural killer cells, macrophages, and myeloid and plasmacytoid dendritic cells. During the pSS evolution, as the severity of inflammation increases, the proportion of B cells in the inflammatory infiltrate also increases [6].

Activated lymphocytes from inflammatory infiltrate produce a large amount of beta-2-microglobulin (B2M). Β2M is a low-molecular-weight protein expressed on the surface of nucleated cells. In normal circumstances, this protein is released into body fluids at a constant rate. The serum level of B2M has been established as a prognostic marker in hematologic disorders, solid organ neoplasms, and rheumatic autoimmune diseases (Sjogren’s syndrome, systemic lupus erythematosus, rheumatoid arthritis). In patients with sicca complex, a high value of serum B2M represents an independent predictor of pSS development. In patients with pSS, the serum B2M is correlated with disease activity and extra-glandular systemic involvement, representing a prognostic factor for lymphoma development [7,8,9,10]. Furthermore, the cells found in the inflammatory infiltrate produce large amounts of cytokines, which are involved in pSS pathogenesis. Previous studies showed that in pSS patients, the levels of IL-1, IL-2, IL-4, IL-5, IL-6, IL-17, IL-21, IL-22, TNF-α, IFN-γ, macrophage colony-stimulating factor (M-CSF), B cell activating factor (BAFF), and mast cell growth factor (MCGF) were increased [11,12,13,14,15,16].

Glandular inflammatory infiltrate is shown by means of histopathological exam. In order to perform this exam, the minor salivary glands, found on the inner lip, are considered the most accessible structures from which to perform biopsy. For the accuracy of this procedure, it is necessary to remove at least four salivary gland lobules, and the Focus score is calculated by an experienced pathologist. A Focus score equal to or higher than 1 represents the cutoff value over which pSS is defined with a sensitivity and specificity of 83.5% and 81.8%, respectively [17,18].

Salivary gland involvement can also be identified through imaging methods. Among them, salivary gland ultrasonography is used as a diagnostic and prognostic tool in pSS, having a comparable sensitivity and specificity to sialography and scintigraphy. It can be performed in grayscale, color/power Doppler, and even ARFI. This method has seen an increase in popularity in recent years, detecting the volume, echogenicity, inhomogeneity, and vascularization of the salivary glands. Over time, many studies regarding the utility of salivary gland ultrasonography in pSS have been published. Numerous scoring systems of salivary glandular involvement in pSS have been developed as a consequence [19,20,21,22].

The histopathological changes in the salivary glands generate a reduction in the salivary flow rate. The Saxon test is a simple, non-invasive method used to detect the salivary flow rate. Some authors consider it the equivalent of the ocular Schirmer’s test [23,24,25].

The main focus of the present study was represented by the evaluation of the salivary flow rate in relation to salivary gland ultrasonographic findings and markers of pSS activity in patients suffering from this disease.

## 2. Results

A total of 112 patients with primary Sjögren’s syndrome (pSS) and 56 healthy controls were included in this study. Table 1 provides a summary of the demographic characteristics for both groups. There was a marked female predominance (66.07% females vs. 33.92% males), with a mean age of 55.53 ± 7.08 years. The average disease duration among pSS patients was 7.53 ± 3.94 years. At the time of the investigation, the medications being used by pSS patients included Hydroxychloroquine (88 patients), Azathioprine (43 patients), Mycophenolate mofetil (45 patients), Cyclophosphamide (12 patients), and corticosteroids (68 patients).

Disease activity was assessed using the EULAR Sjögren’s Syndrome Disease Activity Index (ESSDAI), with a mean score of 12.08 ± 7.53. Based on the ESSDAI scores, patients were categorized as having low disease activity (ESSDAI < 5: 22 patients), moderate disease activity (ESSDAI 5–13: 45 patients), or high disease activity (ESSDAI ≥ 14: 45 patients). The average Focus score was 4.03 ± 1.60.

All pSS patients tested positive for antinuclear antibodies and rheumatoid factor, whereas none of the controls exhibited these markers. Among the antinuclear antibodies, anti-SSA and anti-SSB antibodies were detected in the patient group (anti-SSA: 85.41 ± 56.43 U/mL, anti-SSB: 65.65 ± 54.88 U/mL), but anti-centromere antibodies were not present in any of the pSS patients.

The levels of TNF-α and IL-6 were significantly elevated in the pSS group compared to controls (*p* < 0.0001). Additionally, serum β2-microglobulin, a marker of lymphocyte activity, was significantly higher in pSS patients than in controls (*p* < 0.01). In contrast, leukocyte and lymphocyte counts were significantly lower in the pSS patients (*p* < 0.001). These results are summarized in Table 2.

**Figure 1:**
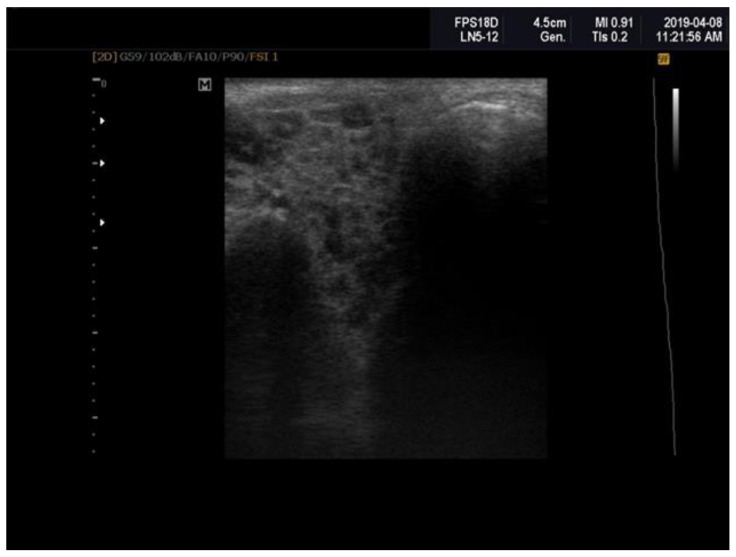
Sjögren’s syndrome; parotid ultrasonography; Hocevar score = 12.

Patients with primary Sjögren’s syndrome (pSS) exhibited notable structural abnormalities in the salivary glands, which were identified through ultrasonographic examination. These abnormalities included reduced parenchymal echogenicity relative to the thyroid gland or surrounding anatomical structures, heterogeneous glandular parenchyma, the presence of hypoechoic areas and hyperechoic foci within the salivary glands, as well as poorly defined glandular borders (Figure 1). The Hocevar SGUS score in pSS patients averaged 26.75 ± 5.91, compared to 5.25 ± 1.08 in the control group, with this difference being statistically significant (*p* < 0.0001).

Due to histological changes in the salivary glands, salivary secretion was reduced in the patients suffering from pSS. The value of Saxon’s test was 1.03 ± 0.33 gm/2 min, in contrast to the controls who registered a value of 6.03 ± 0.33 (*p* < 0.0001).

Correlations between the salivary flow rate (Saxon test), activity biomarkers, histopathological findings (Focus score), and ultrasonography parameters (SGUS) are presented in Table 3. 

**Figure 2:**
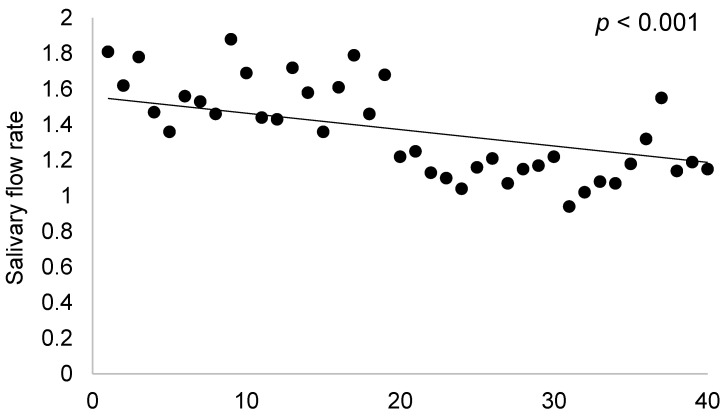
Correlations between salivary flow rate and SGUS.

A strong, negative correlation could be identified between TNF-alpha and salivary flow rate (r = −0.68, *p* < 0.0001), indicating that the higher the values of TNF-alpha, the lower is the salivary flow. Ultrasonography parameters (SGUS) were similarly strong and negatively correlated to salivary flow (r = −68.002, *p* < 0.0001), as can be observed in Figure 2.

Salivary flow was very strongly and inversely correlated to IL-6 levels and Β2-microglobulin, having correlation coefficients of r = −0.78 and r = −0.84, respectively, which reached statistical significance (*p* < 0.0001 in both instances). We can conclude, then, that salivary flow decreases as IL-6 and Β2-microglobulin levels increase.

The strongest negative correlations of salivary flow rate were those with the Focus score (r = −0.82, *p* < 0.0001) and ESSDAI score (r = −0.88, *p* < 0.0001), indicating that salivary flow rate is lower in individuals with higher ESSDAI and Focus scores.

Other correlations established between ultrasonographic abnormalities (SGUS), activity biomarkers, and histopathological parameters are summarized in Table 4.

In patients in which the SGUS findings showed modifications, a strong inverse correlation was observed with salivary flow, signifying that salivary flow is decreased in patients with modified SGUS (r = −0.68, *p* < 0.0001).

IL-6 and Β2-microglobulin levels were positively and strongly correlated to the SGUS findings, having correlation coefficients of 0.735 and 0.734, respectively, which were statistically significant (*p* < 0.0001). Patients with more significant SGUS findings also had higher IL-6 and Β2-microglobulin levels.

The SGUS findings were also strongly and positively correlated with the ESSDAI and Focus scores (r = −0.79 and r = −0.76, *p* < 0.0001), revealing that in patients with higher score values, the ultrasonography findings are more numerous.

## 3. Discussion

The present study showed the existence and degree of correlations between decreased salivary flow and activity markers and ultrasonographic scores in pSS patients.

SS is a multi-systemic autoimmune disease, characterized by a chronic lymphocytic infiltration of the exocrine glands, mainly the salivary and lacrimal ones [26]. Aside from the glandular involvement, due to the systemic nature of SS, almost every organ can be affected [27]. Depending on the associated conditions, SS can be primary (pSS, not associated with other diseases) or secondary (associated with rheumatological or infectious diseases) [4]. pSS has a slow progression and is a non-life-threatening disease with a 10-year cumulative survival rate of over 90%, though some patients develop a severe form of disease and have an increased mortality risk [28].

Nowadays, pSS diagnosis is established based on a combination of clinical, serological, histological, functional, and instrumental parameters, which detect systemic, salivary gland, and lacrimal gland modifications [29]. Salivary gland injury induced by the immune-mediated response, B-cell hyperactivation, and inflammatory glandular infiltration leads to a reduced salivary flow rate, resulting in xerostomia. This represents one of the main complaints of pSS patients. Active glandular inflammation (infiltration of immune cells) and chronic damage (fibrosis and fatty lesions) generate the loss of functional parenchyma. Functional impairment, represented by autonomic dysfunction and receptor-mediated downregulation of saliva, contributes to hyposalivation in these patients [30,31].

All of the studied pSS patients presented xerostomia of different degrees, without it being present in any of the controls. Salivary flow decreased with time. This study showed significant correlations between disease duration and stimulated salivary flow (Saxon test), as well as SGUS (*p* < 0.05). The same results were reported by Pijpe J et al. in their study. The authors demonstrated that patients with Sjögren’s syndrome with a longer disease duration presented important reduced salivary secretions [32].

Many types of cells are involved in the pathogenesis of pSS. They (T cells: Th17 and Th22 cells, B cells, follicular dendritic cells, and innate immune system cells) interact with each other, generating glandular inflammation, which has a persistent character, causing structural and functional glandular changes [33,34]. These cells secrete a series of autoantibodies and inflammatory mediators, which increase glandular inflammation [35,36]. Some of these antibodies can be detected years before the first clinical signs are present in these patients. Theander et al. showed in their study that anti-SSA and anti-SSB antibodies were identified in 81% of 117 pSS patients even in the absence of any sign of disease [37]. The pSS patients in this study presented high levels of anti-SSA (85.41 ± 56.43 units/mL) and anti-SSB (65.65 ± 54.88 units/mL) antibodies. They presented higher levels of circulating cytokines (TNF-α and IL-6) than those found in the control group, reaching a statistically significant threshold (*p* < 0.0001). Chen C et al. showed that in pSS patients, there were higher levels of proinflammatory cytokines when compared to the control group (*p* < 0.001). Furthermore, the increased levels of those cytokines were correlated with the ESSDAI score (*p* < 0.05) [35]. Another study revealed that proinflammatory cytokines were present at higher levels in pSS patients than in patients in the control group, and IL-6 correlated with the ClinESSDAI score (*p* = 0.036) [38].

Activated inflammatory cells release B2M into the circulation. Following glomerular filtration, B2M is completely reabsorbed and catabolized in the proximal renal tubules. The high levels of serum and urinary B2M levels are associated with various hematologic malignancies, autoimmune diseases, and renal disorders [39,40,41]. It is known that in patients with sicca complex, serum B2M is an independent predictor of further pSS development [9]. In pSS patients, serum B2M has been associated with extra-glandular systemic manifestations, including the development of lymphoma [10]. The present study revealed that in pSS patients, there were higher levels of B2M (3.39 ± 1.02 mg/L) than in the control group (1.92 ± 0.46 mg/L), with the difference being statistically significant (*p* < 0.01), signifying a markedly increased inflammatory activity in the first group compared to the latter. Similar to our findings, Tecer et al. showed that patients who presented with anti-SSA and anti-SSB antibodies also had significantly higher serum B2M levels than both patients who only presented anti-SSA antibodies or those in whom these autoantibodies were absent [10]. Gottenberg et al. identified that the serum B2M level was significantly correlated with serum RF (r = 0.33, *p* = 0.001), IgG (r = 0.42, *p* = 0.001), and ESR (r = 0.39, *p* = 0.001) [42]. Tecer et al. reported that the ESSDAI score was significantly correlated with serum B2M levels (r = 0.482, *p* = 0.001). In another study, published by Pertovaara and Korpela, the same correlation between serum B2M levels and the ESSDAI score (r = 0.383, *p* = 0.001) was reported [9,10]. These findings underline the link between pSS activity (reflected in the ESSDAI score) and the activation of inflammatory cells producing B2M.

Salivary gland injury caused by the immune-mediated destruction of the exocrine glands is responsible for xerostomia, and presently, this glandular damage can be evaluated by imagistic methods. Among them, salivary gland ultrasound (SGUS) represents a simple, inexpensive, noninvasive, and non-irradiating method [43].

By means of parotid and submandibular gland ultrasonography, glandular abnormalities can be detected: decrease in parenchymal echogenicity compared to the thyroid or to surrounding anatomic structures, heterogeneity of glandular parenchyma, presence of hypoechoic/anechoic areas, hyperechoic bands and hyperechoic foci in salivary glands, fatty infiltration, and difficult visibility of glandular borders [19]. The pSS patients enrolled in this study presented these elements. Over time, several scoring systems of ultrasonographic changes in patients with pSS have been developed [20,21,22,29]. In the present study, Hocevar’s SGUS score was utilized. Its value was approximately 26.75 ± 5.91 in the studied pSS patients, compared to 5.25 ± 1.08 in the control group, with the difference reaching statistical significance (*p* < 0.0001). This SGUS score was correlated with the salivary flow rate, disease activity, and its biomarkers, and all these correlations reached the threshold for statistical significance (*p* < 0.0001).

The glandular inflammatory infiltrate identified by means of histopathological examination also generates ultrasonographic changes. Inflammatory cells, especially lymphocytes, produce antibodies, cytokines, and increased amounts of B2M. Inflammation encourages an increase in pSS activity, reflected in the value of the ESSDAI score. The consequences of glandular destruction and subsequent modifications in the salivary gland lead to a decrease in salivary flow rate [44,45]. These correlations have been supported by many studies. Some of them analyzed the correlations between varying independent parameters, while the present study analyzed the correlations between all these parameters simultaneously.

Abnormal SGUS was associated with higher ESSDAI activity scores (*p* < 0.001), higher IgG values (*p* < 0.001), serum mono- and oligoclonal bands (*p* = 0.02), and severe modifications shown in biopsies (including the development of germinal centers) (*p* = 0.007) [43]. Analyzing 105 pSS patients, Zhang et al. demonstrated that the SGUS scores were associated with inflammatory and immune activity (*p* < 0.05) [46]. In a group of 70 pSS patients, Fidelix et al. described that low SGUS scores were associated with an ESSDAI < 5, while high scores were associated with an ESSDAI ≥ 5, indicating that high scores were correlated with a lower salivary flow rate (*p* = 0.001) [47]. Lee et al. concluded that the SGUS score was correlated with the unstimulated salivary flow rate, serum rheumatoid factor, and IgG (*p* < 0.001) [48]. Studying 303 patients with pSS, Milic et al. found that patients with moderate and high ESSDAI scores had significantly higher ultrasound scores compared to that of pSS patients with low disease activity (*p* = 0.006 and *p* = 0.01, respectively). The authors concluded that the SGUS could represent a surrogate marker of disease activity and damage progression [49]. The authors showed that homogeneity was an independent risk factor for a low unstimulated salivary flow rate in these patients, based on the logistic regression analysis (OR 1.586, *p* = 0.001) [50]. A relationship between the SGUS score, salivary flow rate, and pSS activity was described by Inanc et al. High SGUS scores were associated with a high disease activity index (*p* = 0.013) and low salivary flow rate (*p* = 0.003) [51]. In their study on salivary glands, Zandonella Callengher et al. used the early 1992 De Vita et al. score and the latest 2019 OMERACT score. They were semiquantitative scoring systems focused on ultrasonographic parenchymal inhomogeneity (grades 0 and 1 being normal, grades 2 and 3 being pathological). The authors found that grades 0 and 1 were associated with rheumatoid factor absence (*p* = 0.002), as well as with a low value or even the absence of serum monoclonal component (*p* = 0.003). Furthermore, these patients presented low disease activity (ESSDAI < 5) (*p* = 0.03) and negative lip biopsy (*p* = 0.029) [52]. Morphological SGUS modifications were associated with a reduced salivary flow rate (*p* < 0.05), hypergammaglobulinemia (*p* < 0.05), and higher Focus score (*p* < 0.05) [53]. Zhang et al. analyzed SGUS in 246 pSS patients, 140 control subjects with other conditions than pSS, and 27 healthy controls. The SGUS scores were higher in the pSS patients than in the non-pSS group, a result that reached statistical significance (*p* < 0.001). Patients with pSS and high SGUS scores had a longer disease duration and presented parotid enlargement and higher levels of serological markers (*p* < 0.001) [54]. Studying the correlations between the histopathological findings, SGUS scores, and serological levels, Delli et al. discovered that the combined usage of SGUS and serology had high predictive value for pSS diagnosis [55].

The small sample size was one limitation of our study, though given the incidence and prevalence of this disease in the general population, it is expected and understandable that the sample size identified in a county would not be one of impressive proportions. Though the present study is a single-center one, it is our hope and aim to be able to establish a network of centers that coordinate in such clinical studies in the future. In order to further refine the findings of the present study, our team is currently working to collect more data and follow the evolution of these patients under treatment.

## 4. Materials and Methods

### 4.1. Patient Selection

The present study has been conducted at the Railway Clinical Hospital, in the Rheumatology Division of the Internal Medicine Department, in Timișoara, Romania, from September 2018 to July 2023. A total of 112 consecutive patients diagnosed with primary Sjögren’s syndrome (pSS) were included, alongside 56 healthy age- and sex-matched controls. All patients with pSS met the 2016 ACR/EULAR classification criteria for primary Sjögren’s syndrome.

The classification criteria are based on the weighted sum of five items: anti-SSA/Ro antibody positivity and focal lymphocytic sialadenitis with a focus score of ≥1 foci/4 mm^2^ (each scoring 3); an ocular staining score of ≥5 (or van Bijsterveld score of ≥4); a Schirmer’s test result of ≤5 mm/5 min; and an unstimulated salivary flow rate of ≤0.1 mL/min (each scoring 1). Individuals with signs and/or symptoms suggestive of Sjogren’s syndrome who had a total score of ≥4 for the above items were considered to have met the criteria for PpSS [56].

The exclusion criteria for this study included patients under 18 years of age, those with secondary Sjögren’s syndrome, overlap syndromes, or sicca symptoms due to other conditions such as sarcoidosis, amyloidosis, IgG4-related disease, hepatic infection with hepatitis C virus (HCV), acquired immunodeficiency syndrome, radiation therapy previously performed in the head or neck regions, graft-versus-host disease, diabetes mellitus, or acute inflammatory pulmonary diseases within 30 days prior to the investigation. Additionally, patients with chronic pulmonary diseases, pregnant or breastfeeding women, individuals with chronic kidney disease (eGFR < 60 mL/min/1.73 m^2^), individuals with an active smoker status, and those taking medications that could impair salivary gland function were excluded. The control group consisted of healthy individuals admitted to the Internal Medicine Department for routine health check-ups. All participants, both pSS patients and controls, provided written informed consent prior to enrollment. This study adhered to the principles of the Declaration of Helsinki and was approved by the Ethics Committee of the Railway Clinical Hospital, Timișoara, Romania (approval number 482/September 2018).

A comprehensive medical history was obtained from all study participants, along with details regarding their current treatment protocols. Each patient also underwent a thorough clinical examination at the start of the study. Disease activity in pSS was assessed using the EULAR Sjögren’s Syndrome Disease Activity Index (ESSDAI), which evaluates 12 domains: constitutional, lymphadenopathy, glandular, articular, cutaneous, pulmonary, renal, muscular, peripheral nervous system, central nervous system, hematological, and biological. An ESSDAI score of less than 5 indicates low disease activity, scores between 5 and 13 denote moderate activity, and values above 14 signify high disease activity.

In patients with pSS, antinuclear antibodies, anti-SSA, anti-SSB, anti-centromere antibodies, rheumatoid factor, serum beta-2 microglobulin, TNF-α, IL-6, leukocytes, and lymphocytes were measured. The control group was tested for serum beta-2 microglobulin, TNF-α, IL-6, leukocytes, and lymphocytes. Antinuclear antibodies were identified through indirect immunofluorescence (HELMED), while anti-SSA, anti-SSB, and anti-centromere antibodies were detected using a fluoroimmunoenzymatic assay. Rheumatoid factor was assessed via latex agglutination. Serum beta-2 microglobulin was measured by an immuno-enzymatic assay with chemiluminescence detection (CLIA-serum, detection limit: 24.7 pg/mL). The cytokines TNF-α and IL-6 were quantified using chemiluminescence (CLIA-serum, detection limit: 1.7 pg/mL) and electrochemiluminescence (ECLIA-serum, detection limit: 0.04 pg/mL) methods, respectively.

### 4.2. Focus Score Assessment

The Focus score is a key element in the classification criteria for primary Sjögren’s syndrome, as established by the American College of Rheumatology (ACR) and the European League Against Rheumatism (EULAR). It is derived from a histopathological examination of the minor salivary glands, which are easily accessible for biopsy from the inner surface of the lower lip. Following the administration of local anesthesia using 10% Lidocaine, 5–7 minor salivary glands were excised. Hematoxylin–eosin staining was utilized to visualize lymphocytic aggregates, or foci, consisting of 50 or more lymphocytes. The Focus score was determined by the density of these foci per 4 mm^2^ of tissue, with a score of ≥1 considered positive [57].

### 4.3. Salivary Gland Ultrasonography (SGUS)

SGUS was performed using a Siemens ACUSON A2000 system (Munich, Germany) equipped with a multifrequency linear transducer operating at 5–14 MHz. Three independent investigators conducted the examinations, achieving an intra-class correlation coefficient (ICC) of 0.9328.

Initially, a B-mode ultrasound (Fujifilm Arietta 65, Germany) was employed to assess the four major salivary glands (bilateral parotid and submandibular glands) in both longitudinal and transverse planes. Salivary gland echogenicity was compared to thyroid echogenicity. For the parotid glands, scanning was performed in the retromandibular fossa, located anterior to the ear and sternocleidomastoid muscle. For the submandibular glands, evaluation was performed with the patient in a supine position.

The Hocevar SGUS scoring system, ranging from 0 to 48, was applied. Key parameters assessed included parenchymal echogenicity in comparison to thyroid tissue (scored as 0 or 1), glandular homogeneity (scored 0–3), the presence of hypoechoic areas in the glandular tissue (scored 0–3), hyperechoic foci in both the parotid (scored 0–3) and submandibular glands (scored 0–1), and the clarity of glandular borders (scored 0–3). The total SGUS score was calculated by summing the scores across these parameters for all four glands [20].

### 4.4. Investigation of Salivary Flow Rate

Salivary flow rate was assessed by means of Saxon’s test. Both pSS patients and patients in the control group were instructed to abstain from eating, drinking, smoking, or rinsing their mouths for at least one hour prior to the test. The test involved chewing on a 5 × 5 cm dry gauze sponge for 2 min to stimulate saliva production. The weight of the sponge was recorded before and after the test, with the difference between the two measurements representing the amount of saliva produced. A pathological result was defined as salivary production of less than 2.75 g over the 2 min period [23].

### 4.5. Statistical Analysis

The Kolmogorov–Smirnov test was used to verify the normality of data distribution. Normally distributed data are expressed as the mean ± standard deviation. Statistical analysis was performed using parametric tests, including ANOVA and Pearson’s correlation. The results were considered statistically significant when the *p*-value was less than 0.05.

## 5. Conclusions

In Sjögren’s Syndrome, there is a strong correlation between disease activity, serum markers, histopathological exam, and salivary gland ultrasound that can be utilized to the patient’s advantage. SGUS represents a non-invasive, painless means of assessing the state of the salivary glands and, implicitly, the salivary flow of patients, offering valuable insights into disease progression and steps that can be taken in order to improve the patients’ quality of life as much as possible.

## Figures and Tables

**Table 1:** Demographic characteristics of studied patients and control group.

Parameter	pSS Patients	Control Group	*p* Value
Number of patients	112	56	0.187
	Females 74 (66.07%)	Females 36 (64.28%)	
	Males 38 (33.92%)	Males 20 (35.71%)	
Mean age (years)	55.5 ± 7.1	54.2 ± 5.9	0.197
Disease duration (years)	7.6 ± 3.9	-	-
Medication used by SS patients		-	-
Hydroxychloroquine	88 patients		
Azathioprine	43 patients		
Mycophenolate mofetil	45 patients		
Cyclophosphamide	12 patients		
Corticoids	68 patients		

**Table 2:** Laboratory findings in pSS patients and control group.

Parameter	pSS Patients	Control Group	*p*-Value
Focus score	4.03 ± 1.60	-	-
ESSDAI	12.08 ± 7.53	-	-
Anti-SSA abs.	85.41 ± 56.43	-	-
Anti-SSB abs.	65.65 ± 54.88	-	-
TNF-α (pg/mL)	27.82± 17.78	7.80 ± 4.74	<0.001
IL6 (pg/mL)	32.68 ± 15.14	6.79 ± 5.12	<0.001
β2-Microglobulin (mg/L)	3.39 ± 1.02	1.92 ± 0.46	<0.01
Leukocytes/mmc	3083.75 ± 1101.01	7586.94 ± 1227.08	<0.001
Lymphocytes/mmc	749.56 ± 368.51	2224.01 ± 419.34	<0.001

**Table 3:** Correlations between salivary flow rate and activity biomarkers, Focus score, SGUS, and ESSDAI.

Parameter	r	*p*
TNF-α	−0.68126	<0.001
IL-6	−0.78466	<0.001
Β2-Microglobulin	−0.84076	<0.001
Focus score	−0.82271	<0.001
SGUS	−0.68002	<0.001
ESSDAI	−0.88205	<0.001

**Table 4:** Correlations between SGUS and salivary flow rate, activity biomarkers, ESSDAI, and Focus scores.

Parameter	r	*p*
Salivary flow rate	−0.68002	<0.001
TNF-α	0.59253	<0.001
IL-6	0.73536	<0.001
β2-microglobulin	0.73417	<0.001
Focus score	0.76661	<0.001
ESSDAI	0.79405	<0.001

## Data Availability

The data are contained within the article.
